# Fatty Placenta, Reply to Letter

**Published:** 1857-06

**Authors:** D. L. M’Gugin

**Affiliations:** Keokuk, Iowa


The letter publised below, handed to us by Prof. McGugin, we gladly insert, and hope it may meet the eye and prove satisfactory to the writer of the letter referred to.—ED.

KEOKUK, IOWA, April 14, 1857.

*Messrs. Editors.*

During my protracted illness, I received a letter from a medical gentleman, dated I think, Plymouth, Illinois, but as the letter was mislaid, I cannot refer to it now.

The subject matter was in relation to a fatty placenta which had then recently fallen under his notice, and the writer desired our views on the subject, if I remember rightly, of its frequency, and cause of its production.

Instances of steroid placenta were observed more than a century ago, by Haller and one or two others, since which it has been frequently seen by a number of pathologists, but its importance, says Simpson, was not so much considered until a similar deposition was found in other organs. Barnes found it to be connected with the fœtus, and that the deposits were found in the structure composing the placental tufts. Since then, according to Dr. Simpson, Bennett has been prosecuting some enquiries into its nature, and believes, in many cases, the fatty degenerations to have been exudations, first thrown out as the result of an inflammatory condition of the structures, in which opinion Priestly concurs. Stein found white masses like fat, or cartilage, but according to the author above referred to, these were supposed to have been coagula thrown out by congestion and hemorrhage.

The death of the fœtus, and the subsequent retention of the placenta, are circumstances which have been found to coexist, from which facts, an inference has been drawn, but erroneously it is believed, that the morbid change in the placenta was the cause of the death of the former. We can readily suppose that a morbid change would result to the placenta when the circulation would cease in the fœtus. But I am not satisfied that this is by any means the case in a majority of instances. If, as it is believed, fatty degeneration takes place most frequently as a consequence of placentitis, is it not reasonable to suppose that, where coaguable lymph has been thrown out, and induration following as a consequence, the circulation in the placental tufts would be altered and diminished, and if so, would not the child suffer and die when not sufficiently nourished? That there is congestion of the placenta, Dr. Simpson admits, but this may take place either from an increased momentum of maternal blood to the placenta, or some interruption of the normal quantity to the child, thereby favoring accumulation. In either, or any of the primary morbid changes from a normal state of the placenta, the fœtus may suffer because of the diminished supply of blood. Some alteration in the umbilical cord which would not allow the transit of the blood through it, may have a backward action on the placenta by engorging it, and a fatal forward effect upon the fœtus.

With the hope that this may meet the eye of the gentleman who addressed us the letter alluded to, I subscribe myself, Yours, &c., D. L. M'GUGIN.

